# Identification of Target Genes at Juvenile Idiopathic Arthritis GWAS Loci in Human Neutrophils

**DOI:** 10.3389/fgene.2019.00181

**Published:** 2019-03-27

**Authors:** Junyi Li, Xiucheng Yuan, Michael E. March, Xueming Yao, Yan Sun, Xiao Chang, Hakon Hakonarson, Qianghua Xia, Xinyi Meng, Jin Li

**Affiliations:** ^1^Department of Cell Biology, 2011 Collaborative Innovation Center of Tianjin for Medical Epigenetics, Tianjin Key Laboratory of Medical Epigenetics, Tianjin Medical University, Tianjin, China; ^2^Center for Applied Genomics, Children’s Hospital of Philadelphia, Philadelphia, PA, United States; ^3^Division of Human Genetics, Children’s Hospital of Philadelphia, Philadelphia, PA, United States; ^4^Department of Pediatrics, Perelman School of Medicine, University of Pennsylvania, Philadelphia, PA, United States

**Keywords:** juvenile idiopathic arthritis, target gene identification, epigenetic regulation, protein-protein interaction, pathway enrichment

## Abstract

Juvenile idiopathic arthritis (JIA) is the most common chronic rheumatic disease among children which could cause severe disability. Genomic studies have discovered substantial number of risk loci for JIA, however, the mechanism of how these loci affect JIA development is not fully understood. Neutrophil is an important cell type involved in autoimmune diseases. To better understand the biological function of genetic loci in neutrophils during JIA development, we took an integrated multi-omics approach to identify target genes at JIA risk loci in neutrophils and constructed a protein-protein interaction network via a machine learning approach. We identified genes likely to be JIA risk loci targeted genes in neutrophils which could contribute to JIA development.

## Introduction

Juvenile idiopathic arthritis (JIA) is the most common chronic rheumatic disease in childhood at a prevalence rate of 1 in 1000, and JIA is a common cause of disability among children (Oen and Cheang, 1996). The typical clinical manifestation of JIA is joint enlargement of unknown origin for more than 6 weeks in children under 16 years old (Petty et al., 2004). JIA has long been considered as a type of autoimmune disease, however, its etiology is still not fully understood. Similar to other complex diseases, genetic and environmental factors both contribute to its pathogenesis (Glass and Giannini, 1999). Substantial evidence suggests the large contribution of genetic components. Previous studies showed that monozygotic twin concordance rates for JIA are between 25 and 40%, much higher than the population prevalence rate (Savolainen et al., 2000). Affected sibling studies showed that siblings of JIA probands had an over 10-fold increased risk of developing the disease (Frisell et al., 2016). Our recent heritability study based on SNP-h2 estimated that the heritability of JIA is 0.73 among the most highly heritable pediatric autoimmune diseases (Li et al., 2015b). Several genome-wide association studies (GWAS) have been carried out and discovered a number of JIA susceptibility loci, but how these loci affect the pathogenesis and development of JIA remains to be explored (Behrens et al., 2008; Hinks et al., 2009, 2013; Thompson et al., 2012; Cobb et al., 2014; Aydin-Son et al., 2015; Li et al., 2015a; Finkel et al., 2016; Ombrello et al., 2017; Haasnoot et al., 2018).

Neutrophils are one of the most important innate immune cells in human bodies. When infection or inflammation occurs, they are recruited to the disease site under the attraction of chemokines. In recent years, studies have found that neutrophils can secrete a variety of cytokines to play a key role in immunomodulation. The clinical manifestations of JIA are highly similar to those of classical autoinflammatory diseases. The large accumulation of white blood cells is one of the causes for local tissue damage and loss of joint function due to the inflammatory response at the joint (Fattori et al., 2016). Neutrophils likely play an important role in the effector phase of autoimmune diseases including JIA, and their action can cause or exacerbate articular inflammation (Németh and Mócsai, 2012). Neutrophil extracellular traps (NETs) are the newly discovered mechanism by which neutrophils fight infection, and has been demonstrated to play a role in pathogenesis of systemic immune diseases such as systemic lupus erythematosus (SLE) (Hakkim et al., 2010), antineutrophil cytoplasmic antibodies (ANCA)-associated systemic vasculitis (Kessenbrock et al., 2009) and multiple sclerosis (Naegele et al., 2012). However, little is known about the genes involved in JIA development in neutrophils.

A number of JIA loci have been identified in GWAS (Behrens et al., 2008; Hinks et al., 2009, 2013; Thompson et al., 2012; Cobb et al., 2014; Aydin-Son et al., 2015; Li et al., 2015a; Finkel et al., 2016; Ombrello et al., 2017; Haasnoot et al., 2018), but few have been functionally characterized as most of the GWAS SNPs are located at the intronic or intergenic regions, without directly affecting the sequence of any protein product. We hypothesize that they may function as *cis-*regulatory elements, regulating target gene expression. Therefore, we focused on understanding the target genes of JIA GWAS loci in neutrophils.

JIA is a heterogeneous group of diseases including several different subtypes. In recent years, due to the progress in disease management, their prognosis has been greatly improved, but there are still few effective treatments. Our study took an *in silico* analysis approach, utilizing genomics, transcriptome, epigenome, and methylome data to find genes targeted by JIA risk loci in neutrophils, facilitating the design of precision strategy of JIA prevention and treatment.

## Materials and Methods

### Extraction of JIA GWAS Loci

Juvenile idiopathic arthritis loci identified in previous GWAS were found in GWAS catalog (MacArthur et al., 2017) by conducting search using keyword “Juvenile idiopathic arthritis.” All loci found were downloaded without further imposing any significance threshold.

### eQTL Analysis

eQTL analysis was performed via Genotype-Tissue Expression (GTEx) Project website (Lonsdale et al., 2013), from which the correlation between each SNP genotype and gene expression level in whole blood was extracted. We set the significance threshold as *P*-value < 0.05. The boxplots for the SNP-gene pairs were reviewed via GTEx Portal.

### Analysis of Microarray Data

Series matrix files of microarray datasets GSE11083 (Frank et al., 2009a,b) and GSE67596 (Jiang et al., 2015) containing transcriptome data from neutrophils of 36 JIA patients and 26 healthy controls were downloaded from NCBI Gene Expression Omnibus (GEO) (Edgar et al., 2002; Barrett et al., 2013). Gene expression levels were compared between JIA patient group and control group. Expression values across studies was summarized through median polish and normalization was performed using Robust Multi-array Average (RMA) algorithm which minimizes variance across arrays and log transformation was conducted for variance stabilization (Irizarry et al., 2003). Meta-analysis were performed using RankProd package (Hong et al., 2006) in R 3.5.1 (R Core Team, 2018). The threshold used to select for differentially expressed genes was defined as possibility of false positives (PFP) < 0.05 and absolute value of fold change (FC) > 1.2.

### Histone Modification Analysis

The SNPs of interest were input into web portal Haploreg^1^ (Ward and Kellis, 2012) and their overlap with histone modification regions in neutrophil cell line E030 BLD.CD15.PC (primary neutrophils from peripheral blood) was evaluated using epigenome data from ROADMAP epigenomics database (Kundaje et al., 2015).

### Methylation Data Analysis

The methylation data were extracted from the genome-wide methylation profiles of 843 subjects processed on the Infinium HumanMethylation450 BeadChip at the Center for Applied Genomics, the Children’s Hospital of Philadelphia, which has been described in previous publication (Van Ingen et al., 2016). The log2 ratio between the methylated and unmethylated intensities of each probe on the chip was represented by the *M*-values. The association between JIA SNP genotype and methylation probes in each of the 11 genes was assessed in a linear regression model conditioned on gender, age and 10 genotype-derived principle components.

### Construction of Protein-Protein Interaction (PPI) Network

Protein-Protein Interaction network on the 11 target genes was constructed via NetworkAnalyst^2^ (Xia et al., 2014) which was based on integration of machine learning and Walktrap algorithms (Pons and Latapy, 2005). The resource of protein-protein interaction data was IMEx Interactome database (Orchard et al., 2012). Hypergeometric test for gene set enrichment analysis was implemented in NetworkAnalyst and the Kyoto Encyclopedia of Genes and Genomes (KEGG) database (Kanehisa et al., 2017) was used as the pathway database resource. In addition to FDR *P*-value calculated based on hypergeometric test and multiple-testing adjustment, empirical *P*-value of pathways was derived from permutation analysis. A list of 11 genes was randomly generated from the human genome and such resampling was performed 100 times. For each 11-gene list randomly drawn, the steps of network construction, pathway analysis were similarly performed as for JIA target genes in neutrophils, and a list of significantly enriched pathways with FDR < 0.05 was resulted from each resampling. For each enriched pathway in PPI network of JIA target genes, its empirical *P*-value was derived based on the number of times it appears as significantly enriched pathway from 100 permutations.

### Hi-C Data Visualization

Hi-C data visualization for the JIA loci and target genes were carried out via the 3D Genome browser^3^ (Wang et al., 2018) and FUMA GWAS^4^ (Watanabe et al., 2017). Hi-C data from cell line K562 (Rao et al., 2014; Schmitt et al., 2016) were used.

## Results

A large number of GWAS loci have been identified for human complex diseases, including JIA. We extracted all 127 genomic regions that have been reported to be associated with JIA from GWAS catalog (Behrens et al., 2008; Hinks et al., 2009, 2013; Thompson et al., 2012; Cobb et al., 2014; Aydin-Son et al., 2015; Li et al., 2015a; Finkel et al., 2016; MacArthur et al., 2017; Ombrello et al., 2017; Haasnoot et al., 2018). All these SNPs are located outside of gene exons, which may contribute to disease etiology by affecting gene expression. We then input these SNPs into GTEx database (Lonsdale et al., 2013) to identify genes that are regulated by these SNPs. Because GWAS SNPs and their target genes may not always exhibit highly significant correlation in eQTL analysis, exemplified by obesity SNP rs9930506 and IRX3 gene (Smemo et al., 2014), we set the significance threshold as nominal *P*-value < 0.05. We found that the expression level of 238 genes correlates with JIA SNP genotype in whole blood.

As we are particularly interested in identifying genes regulated by JIA GWAS loci in neutrophils, we examined which of these 238 genes showed differential expression in neutrophils between JIA cases and controls. We extracted two microarray datasets from gene expression omnibus (GEO) database, GSE11083 (Frank et al., 2009a,b) and GSE67596 (Jiang et al., 2015). Gene expression data from a total of 36 JIA cases and 26 controls were meta-analyzed. Among the 264 eQTL genes for JIA SNPs, only 11 genes showed significant differential expression, including 5 up-regulated and 6 down-regulated (Table 1). Our *in silico* analysis suggested that these genes may function as JIA loci targeted genes in neutrophils. Among the 13 pairs of JIA SNPs and target genes, only SNP rs79893749 is located in the intron of its target gene *CCR3*; all the other SNPs are located outside of the transcript region of their target genes. Hi-C data provide additional supporting evidence for plausible chromatin interactions between some JIA SNPs and their target genes (Supplementary Figures 1, 2), with the caveat that these data came from a chronic myelogenous leukemia cell line K562 (Rao et al., 2014; Schmitt et al., 2016). Experiments using neutrophils would be necessary to further explore their possible interactions.

**Table 1:** Summary of JIA GWAS loci targeted genes in neutrophils.

								DEG			Methylation	
SNP	Chr	Pos (hg19)	Gene	Location		GWAS_Pval	GTEx_Pval	PFP	FC	Histone Mark	Probe	Pval
rs4648881	1	25197155	*MTFR1L*	5^′^		5.E-07	4.0E-03	2.33E-02	-1.30	none		


rs9633402	1	247946160	*TRIM58*	5^′^		3.E-06	5.4E-04	1.15E-02	1.21	none	cg12689806	8.45E-02


rs79893749	3	46253650	*CCR3*	intron		2.E-07	2.5E-04	1.68E-02	-1.27	H3K4me1_Enh	cg04111761	7.78E-03


rs4869313	5	96223880	*ELL2*	5^′^		9.E-08	2.4E-02	4.04E-02	-1.26	none		


rs41291794	6	32425762	*HLA-DPA1*	3^′^		^∗^4.E-15	1.4E-02	8.20E-04	1.33	none	cg13906813	1.64E-02


rs7069750	10	90762376	*ACTA2*	5^′^		^∗^3.E-08	4.9E-20	3.60E-03	1.27	none	cg03111039	^∗∗^6.52E-08


rs7069750	10	90762376	*ANKRD22*	5^′^		^∗^3.E-08	4.0E-03	3.64E-02	1.21	none	cg15103050	1.52E-01


rs12598357	16	28340945	*SH2B1*	5^′^		^∗^4.E-09	4.4E-06	3.83E-04	1.68	none	cg07884168	1.55E-02


rs12598357	16	28340945	*SULT1A1*	3^′^		^∗^4.E-09	9.6E-07	5.51E-03	-1.31	none	cg09685060	^∗∗^1.41E-09


rs12928404	16	28847246	*SH2B1*	5^′^		6.E-07	1.9E-04	3.83E-04	1.68	none	cg06932837	^∗∗^5.13E-05


rs12928404	16	28847246	*SULT1A1*	5^′^		6.E-07	5.0E-06	5.51E-03	-1.31	none	cg26603685	^∗∗^1.72E-07


rs2847293	18	12782448	*MPPE1*	5^′^		^∗^1.E-12	3.9E-02	2.01E-02	-1.36	none	cg14599440	2.01E-03


rs149850873	18	12885120	*CEP192*	5^′^		5.E-07	6.0E-03	4.90E-03	-1.37	H3K4me1_Enh H3K4me3_Pro	cg00686761	1.78E-01


*SNP = single nucleotide polymorphism; Chr = chromosome; Pos (hg19) = position on human genome build hg19; Gene = likely targeted gene at each GWAS locus; Location = The SNP is located within intron, 5^′^ upstream or 3^′^ downstream of its target gene. GWAS_Pval = *P*-value of each SNP in GWAS analyses extracted from GWAS catalog; GTEx_Pval = *P*-value of correlation between gene expression level and GWAS SNP genotype in GTEx database; DEG_PFP = the probability of being false positive for the differentially expressed gene in the microarray analysis; DEG_FC = fold change of the differentially expressed gene in a microarray analysis; Histone Mark = histone marks overlap with each SNP in cell line E030 BLD.CD15.PC; Methylation_probe = the methylation probe with the minimum *P*-value in the target gene for association testing with the GWAS SNP; Methylation_Pval = the *P*-value of the methylation probe in methylation analysis; ^∗^GWAS_Pval < 5.E-08; ^∗∗^Methylation *P*-value < 3.5E-04; none = no histone modification was found in cell line E030 BLD.CD15.PC.*

**FIGURE 1:**
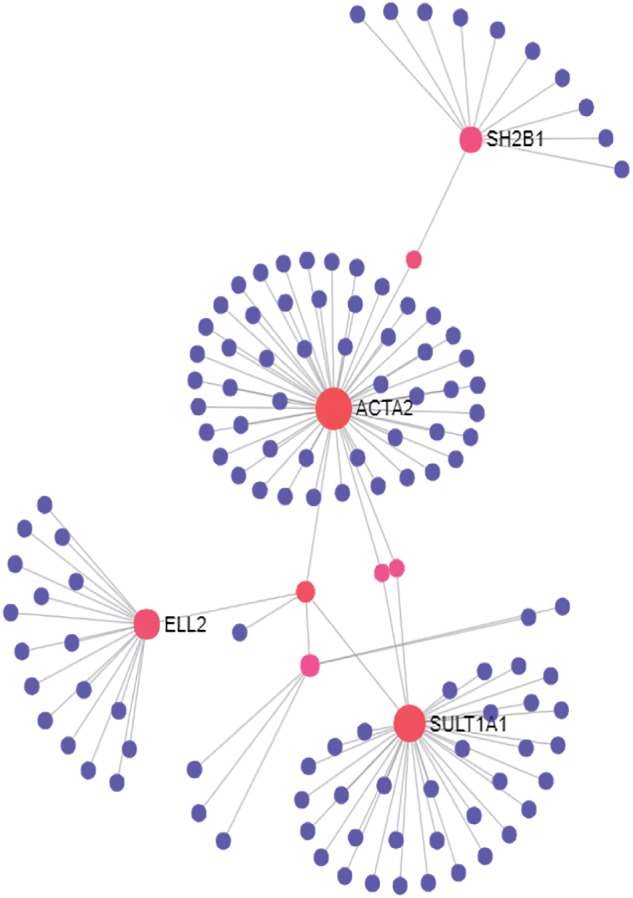
PPI network of first-order interactions constructed for JIA loci target genes in neutrophils.

To understand how these genes coordinately contribute to JIA development, we constructed PPI network among proteins encoded by these genes and their direct interactors (Figure 1) using NetworkAnalyst which integrates statistical analyses and machine learning for interactive PPI network visualization. We further conducted pathway analysis and found several signaling pathways significantly enriched among proteins in this network, including neurotrophin signaling pathway, cardiac muscle contraction, cell cycle and hypertrophic cardiomyopathy (HCM) (Table 2). To test the cell type specificity of 11 target genes and enriched pathways, we repeated the whole process using microarray gene expression data from PBMC samples of the same GEO datasets. We found that among the 11 target genes in neutrophils, one gene (TRIM58) was shared with PBMC (Table 1 and Supplementary Table 1). No enriched pathway was shared between neutrophils and PBMC (Figure 1, Table 2, Supplementary Figure 3, and Supplementary Table 2). To further determine the specificity of the 4 enriched pathways among JIA target genes in neutrophils, we checked the distribution of significantly enriched pathways from 100 randomly generated 11-gene lists and derived the empirical *P*-values for each of the 4 pathways of interest (Table 2). The pathways of cardiac muscle contraction and hypertrophic cardiomyopathy were of empirical *P*-value < 0.01. Based on these two control gene set analyses, we demonstrated the specificity of these target genes and pathways in neutrophils, serving the initial screening purpose for further functional validation.

**Table 2:** KEGG pathways enriched among the PPI network formed by JIA loci target gene and their interactors (FDR < 0.05).

Pathway	Total	Expected	Hits	*P*-value	FDR	Empirical *P*-value
Neurotrophin signaling pathway	123	1.34	10	5.60E-07	1.21E-04	0.1
Cardiac muscle contraction	12	0.131	4	5.81E-06	6.30E-04	<0.01^∗^
Cell cycle	124	1.35	8	4.74E-05	3.43E-03	0.14
Hypertrophic cardiomyopathy	25	0.272	4	1.34E-04	7.25E-03	<0.01^∗^


*Total = the total number of genes in each pathway; Expected = the expected number of genes in each pathway given the number of JIA target genes; Hits = the actual number of JIA target genes falling into each pathway; *P*-value = *P*-value of each pathway in enrichment test; FDR = false discovery rate of each pathway; ^∗^empirical *P*-value < 0.01 based on permutation analysis.*

Next, we investigated how JIA loci may regulate the expression of their targeted genes. To address this question, we examined the ROADMAP database (Kundaje et al., 2015) through HaploReg (Ward and Kellis, 2012). We found rs79893749 and rs149850873 overlap with histone marks in a neutrophil cell line E030 BLD.CD15.PC, suggesting that these loci may regulate their targeted gene expression through histone modifications in the promoter or enhancer region. We also looked into the potential mechanism of DNA methylation. In our methylation analysis, we tested the 10 JIA SNPs against their corresponding one or two genes which each contains ∼11 methylation probes on average. A total of 144 SNP-methylation-probe pairs were tested, thus the multiple-testing adjusted *P*-value cutoff is set at 3.5 × 10^-4^. The correlation between four SNP-methylation-probe pairs reached this experiment-wide significance threshold, suggesting that these JIA SNPs may regulate the expression of their target genes through DNA methylation (Table 1).

## Discussion

In this study, we conducted data mining in existing datasets to gain a better understanding of the molecular mechanism of JIA GWAS loci. By eQTL and transcriptome analyses, we identified 11 genes may be JIA loci target genes in neutrophils. We further built PPI network and found pathways enriched among target genes and their interactors. We also found multiple JIA GWAS SNPs overlap with histone marks and/or correlate with methylation level in their target genes.

We did not observe extensive overlap between JIA eQTL genes in whole blood and genes of differential expression in neutrophils. It possibly resulted from the small sample size in our microarray datasets which did not have enough power to detect certain differentially expressed genes. In addition, JIA eQTL genes may be expressed in cell types other than neutrophils which we are particularly interested in.

Several of the target genes we identified are highly related to the immune system, such as *CCR3*, *ELL2*, and *HLA-DPA1*. Others play a role in cell proliferation, carcinogenesis and/or other biological functions. The Human leukocyte antigen (HLA) gene complex encodes human major histocompatibility complex (MHC), a group of cell-surface proteins playing important roles in the regulation of human immune system. *HLA* genes have been reported to be associated with autoimmune diseases (Sollid, 2017; Kawabata et al., 2018), including rheumatoid arthritis (Onuora, 2015; Smolen et al., 2018) and JIA (Smerdel et al., 2002). The *HLA-DPA1* locus has been particularly linked to ankylosing spondylitis, a type of chronic inflammatory rheumatic disease (Diaz-Pena et al., 2011). As expected, *HLA* genes were also found as target genes in PBMC, suggesting they contribute to pathogenesis of JIA in diverse immune cell types. *CCR3* gene encodes a protein as a member of the G protein-coupled receptor family, responding to the C-C type chemokines. SNP in *CCR3-CCR5* region has been linked to family history of autoimmune disease among children with type I diabetes (Parkkola et al., 2017). It has been reported that *CCR3* expression was increased under rheumatoid arthritis conditions, and it mediated eotaxin-1 induced matrix metalloproteinase (MMP)-9 upregulation in fibroblast-like synoviocyte which may further result in articular damage (Liu et al., 2017). Previous studies have also demonstrated that CCR3 is highly expressed in infiltrated synovial neutrophils of rheumatoid arthritis patients (Hartl et al., 2008). *ELL2* gene encodes Elongation Factor for RNA Polymerase II 2, a component of the super-elongation complex. It functions in immune regulation by affecting IgH alternative processing, Ig secretion and plasma cell differentiation. Missense mutation in *ELL2* gene affects IgA and IgG level associated with multiple myeloma (Swaminathan et al., 2015). Study has shown that ELL2 is expressed in mature neutrophils and its expression is elevated in responses to inflammatory stimuli (Zhang et al., 2004). Our results suggest that these genes may also play a role in neutrophils mediating the effect of JIA risk loci during JIA pathogenesis which should be further investigated by experimental approaches.

The pathways of cardiac muscle contraction and hypertrophic cardiomyopathy are significantly and specifically enriched in PPI network of JIA target genes and their interactors in neutrophils. Multiple studies have reported that patients with rheumatoid arthritis have a higher incidence and mortality of cardiovascular disease (Maradit-Kremers et al., 2005; Voskuyl, 2006; Avina-Zubieta et al., 2008; Georgiadis et al., 2008). Cardiac involvement has similarly been found in JIA patients (Svantesson et al., 1983; Hull, 1988). However, whether JIA increases the long-term risk of cardiovascular disease is still uncertain (Coulson et al., 2013). Our results suggest that JIA and cardiovascular disease may share common underlying molecular mechanism.

High-throughput omics technology provides a wealth of experimental data for disease gene discovery. The multi-omics studies on the interplay between genes, RNA, proteins and small molecules reveal new directions for the research of complex diseases (Bersanelli et al., 2016; Bock et al., 2016). Integration of data from different dimensions of multi-omics data via different analytical approaches facilitates prioritizing genes for efficient functional studies and contributes to the understanding of disease etiology.

## Data Availability

All datasets generated for this study are included in the manuscript and/or the Supplementary Files.

## Author Contributions

JL, XM, and QX conceived and designed the study. JYL, XCY, XMY, YS, and QX performed the analysis. MM, XC, and HH provided the methylation analysis data. JYL wrote the manuscript. JL and XM reviewed and edited the manuscript critically. All authors read and approved the manuscript.

## Conflict of Interest Statement

The authors declare that the research was conducted in the absence of any commercial or financial relationships that could be construed as a potential conflict of interest.

## Footnotes

1 https://pubs.broadinstitute.org/mammals/haploreg/haploreg.php

2 https://www.networkanalyst.ca

3 promoter.bx.psu.edu/hi-c/

4 http://fuma.ctglab.nl/

## References

[B1] Avina-Zubieta J. A. Choi H. K. Sadatsafavi M. Etminan M. Esdaile J. M. Lacaille D. (2008). Risk of cardiovascular mortality in patients with rheumatoid arthritis: a meta-analysis of observational studies. *Arthritis Rheum* 59 1690–1697. 10.1002/art.24092 19035419

[B2] Aydin-Son Y. Batu E. D. Demirkaya E. Bilginer Y. Kasapcopur O. Unsal E. (2015). Systems-level analysis of genome wide association study results for a pilot juvenile idiopathic arthritis family study. *Turk J. Pediatr.* 57 324–333. 27186693

[B3] Barrett T. Wilhite S. E. Ledoux P. Evangelista C. Kim I. F. Tomashevsky M. (2013). NCBI GEO: archive for functional genomics data sets–update. *Nucleic Acids Res.* 41 D991–D995. 10.1093/nar/gks1193 23193258 PMC3531084

[B4] Behrens E. M. Finkel T. H. Bradfield J. P. Kim C. E. Linton L. Casalunovo T. (2008). Association of the TRAF1-C5 locus on chromosome 9 with juvenile idiopathic arthritis. *Arthritis Rheum* 58 2206–2207. 10.1002/art.23603 18576341

[B5] Bersanelli M. Mosca E. Remondini D. Giampieri E. Sala C. Castellani G. (2016). Methods for the integration of multi-omics data: mathematical aspects. *BMC Bioinformatics* 17(Suppl. 2):15. 10.1186/s12859-015-0857-9 26821531 PMC4959355

[B6] Bock C. Farlik M. Sheffield N. C. (2016). Multi-omics of single cells: strategies and applications. *Trends Biotechnol.* 34 605–608. 10.1016/j.tibtech.2016.04.004 27212022 PMC4959511

[B7] Cobb J. Cule E. Moncrieffe H. Hinks A. Ursu S. Patrick F. (2014). Genome-wide data reveal novel genes for methotrexate response in a large cohort of juvenile idiopathic arthritis cases. *Pharmacogenomics J.* 14:356. 10.1038/tpj.2014.3 24709693 PMC4091986

[B8] Coulson E. J. Ng W.-F. Goff I. Foster H. E. (2013). Cardiovascular risk in juvenile idiopathic arthritis. *Rheumatology* 52 1163–1171. 10.1093/rheumatology/ket106 23502074

[B9] Diaz-Pena R. Aransay A. M. Bruges-Armas J. Lopez-Vazquez A. Rodriguez-Ezpeleta N. Mendibil I. (2011). Fine mapping of a major histocompatibility complex in ankylosing spondylitis: association of the HLA-DPA1 and HLA-DPB1 regions. *Arthritis Rheum* 63 3305–3312. 10.1002/art.30555 21769851

[B10] Edgar R. Domrachev M. Lash A. E. (2002). Gene expression omnibus: NCBI gene expression and hybridization array data repository. *Nucleic Acids Res.* 30 207–210. 10.1093/nar/30.1.207 11752295 PMC99122

[B11] Fattori V. Amaral F. A. Verri W. A. Jr. (2016). Neutrophils and arthritis: role in disease and pharmacological perspectives. *Pharmacol. Res.* 112 84–98. 10.1016/j.phrs.2016.01.027 26826283

[B12] Finkel T. H. Li J. Wei Z. Wang W. Zhang H. Behrens E. M. (2016). Variants in CXCR4 associate with juvenile idiopathic arthritis susceptibility. *BMC Med. Genet.* 17:24. 10.1186/s12881-016-0285-3 27005825 PMC4804485

[B13] Frank M. B. Wang S. Aggarwal A. Knowlton N. Jiang K. Chen Y. (2009a). Disease-associated pathophysiologic structures in pediatric rheumatic diseases show characteristics of scale-free networks seen in physiologic systems: implications for pathogenesis and treatment. *BMC Med. Genomics* 2:9. 10.1186/1755-8794-2-9 19236715 PMC2649160

[B14] Frank M. B. Wang S. Aggarwal A. Knowlton N. Kaiyu J. Chen Y. (2009b). *Childhood Onset Rheumatic Disease Gene Expression Profile. GEO.* Available at: https://www.ncbi.nlm.nih.gov/geo/query/acc.cgi?acc=GSE11083

[B15] Frisell T. Hellgren K. Alfredsson L. Raychaudhuri S. Klareskog L. Askling J. (2016). Familial aggregation of arthritis-related diseases in seropositive and seronegative rheumatoid arthritis: a register-based case-control study in Sweden. *Ann. Rheum Dis.* 75 183–189. 10.1136/annrheumdis-2014-206133 25498119 PMC4465879

[B16] Georgiadis A. N. Voulgari P. V. Argyropoulou M. I. Alamanos Y. Elisaf M. Tselepis A. D. (2008). Early treatment reduces the cardiovascular risk factors in newly diagnosed rheumatoid arthritis patients. *Semin. Arthritis Rheum* 38 13–19. 10.1016/j.semarthrit.2007.09.008 18191989

[B17] Glass D. N. Giannini E. H. (1999). Juvenile rheumatoid arthritis as a complex genetic trait. *Arthritis Rheum* 42 2261–2268. 10.1002/1529-0131(199911)42:11<2261::AID-ANR1>3.0.CO;2-P 10555018

[B18] Haasnoot A. J. W. Schilham M. W. Kamphuis S. Hissink Muller P. C. E. Heiligenhaus A. Foell D. (2018). Identification of an amino acid motif in HLA-DRbeta1 that distinguishes uveitis in patients with juvenile idiopathic arthritis. *Arthritis Rheumatol.* 70 1155–1165. 10.1002/art.40484 29513936

[B19] Hakkim A. Furnrohr B. G. Amann K. Laube B. Abed U. A. Brinkmann V. (2010). Impairment of neutrophil extracellular trap degradation is associated with lupus nephritis. *Proc. Natl. Acad. Sci. U.S.A.* 107 9813–9818. 10.1073/pnas.0909927107 20439745 PMC2906830

[B20] Hartl D. Krauss-Etschmann S. Koller B. Hordijk P. L. Kuijpers T. W. Hoffmann F. (2008). Infiltrated neutrophils acquire novel chemokine receptor expression and chemokine responsiveness in chronic inflammatory lung diseases. *J. Immunol.* 181 8053–8067. 10.4049/jimmunol.181.11.8053 19017998

[B21] Hinks A. Barton A. Shephard N. Eyre S. Bowes J. Cargill M. (2009). Identification of a novel susceptibility locus for juvenile idiopathic arthritis by genome-wide association analysis. *Arthritis Rheum* 60 258–263. 10.1002/art.24179 19116933 PMC3001111

[B22] Hinks A. Cobb J. Marion M. C. Prahalad S. Sudman M. Bowes J. (2013). Dense genotyping of immune-related disease regions identifies 14 new susceptibility loci for juvenile idiopathic arthritis. *Nat. Genet.* 45 664–669. 10.1038/ng.2614 23603761 PMC3673707

[B23] Hong F. Breitling R. Mcentee C. W. Wittner B. S. Nemhauser J. L. Chory J. (2006). RankProd: a bioconductor package for detecting differentially expressed genes in meta-analysis. *Bioinformatics* 22 2825–2827. 10.1093/bioinformatics/btl476 16982708

[B24] Hull R. G. (1988). Outcome in juvenile arthritis. *Br. J. Rheumatol.* 27(Suppl. 1), 66–71. 3277687

[B25] Irizarry R. A. Hobbs B. Collin F. Beazer-Barclay Y. D. Antonellis K. J. Scherf U. (2003). Exploration, normalization, and summaries of high density oligonucleotide array probe level data. *Biostatistics* 4 249–264. 10.1093/biostatistics/4.2.249 12925520

[B26] Jiang K. Frank M. B. Osban J. Chen Y. Hartman C. Jarvis J. N. (2015). *Common Immunologic Pathways Between Oligoarticular and Polyarticular Juvenile Idiopathic Arthritis. GEO.* Available at: https://www.ncbi.nlm.nih.gov/geo/query/acc.cgi?acc=GSE67596

[B27] Kanehisa M. Furumichi M. Tanabe M. Sato Y. Morishima K. (2017). KEGG: new perspectives on genomes, pathways, diseases and drugs. *Nucleic Acids Res.* 45 D353–D361. 10.1093/nar/gkw1092 27899662 PMC5210567

[B28] Kawabata Y. Nishida N. Awata T. Kawasaki E. Imagawa A. Shimada A. (2018). A genome-wide association study confirming a strong effect of HLA and identifying variants in CSAD/lnc-ITGB7-1 on chromosome 12q13.13 associated with susceptibility to fulminant type 1 diabetes. *Diabetes* 68 665–675. 10.2337/db18-0314 30552108

[B29] Kessenbrock K. Krumbholz M. Schonermarck U. Back W. Gross W. L. Werb Z. (2009). Netting neutrophils in autoimmune small-vessel vasculitis. *Nat. Med.* 15 623–625. 10.1038/nm.1959 19448636 PMC2760083

[B30] Kundaje A. Meuleman W. Ernst J. Bilenky M. Yen A. Heravi-Moussavi A. (2015). Integrative analysis of 111 reference human epigenomes. *Nature* 518 317–330. 10.1038/nature14248 25693563 PMC4530010

[B31] Li Y. R. Li J. Zhao S. D. Bradfield J. P. Mentch F. D. Maggadottir S. M. (2015a). Meta-analysis of shared genetic architecture across ten pediatric autoimmune diseases. *Nat. Med.* 21 1018–1027. 10.1038/nm.3933 26301688 PMC4863040

[B32] Li Y. R. Zhao S. D. Li J. Bradfield J. P. Mohebnasab M. Steel L. (2015b). Genetic sharing and heritability of paediatric age of onset autoimmune diseases. *Nat. Commun.* 6:8442. 10.1038/ncomms9442 26450413 PMC4633631

[B33] Liu X. Zhang H. Chang X. Shen J. Zheng W. Xu Y. (2017). Upregulated expression of CCR3 in rheumatoid arthritis and CCR3-dependent activation of fibroblast-like synoviocytes. *Cell Biol. Toxicol.* 33 15–26. 10.1007/s10565-016-9356-7 27495116

[B34] Lonsdale J. Thomas J. Salvatore M. Phillips R. Lo E. Shad S. (2013). The genotype-tissue expression (GTEx) project. *Nat. Genet.* 45:580. 10.1038/ng.2653 23715323 PMC4010069

[B35] MacArthur J. Bowler E. Cerezo M. Gil L. Hall P. Hastings E. (2017). The new NHGRI-EBI Catalog of published genome-wide association studies (GWAS Catalog). *Nucleic Acids Res.* 45 D896–D901. 10.1093/nar/gkw1133 27899670 PMC5210590

[B36] Maradit-Kremers H. Nicola P. J. Crowson C. S. Ballman K. V. Gabriel S. E. (2005). Cardiovascular death in rheumatoid arthritis: a population-based study. *Arthritis Rheum* 52 722–732. 10.1002/art.20878 15751097

[B37] Naegele M. Tillack K. Reinhardt S. Schippling S. Martin R. Sospedra M. (2012). Neutrophils in multiple sclerosis are characterized by a primed phenotype. *J. Neuroimmunol.* 242 60–71. 10.1016/j.jneuroim.2011.11.009 22169406

[B38] Németh T. Mócsai A. (2012). The role of neutrophils in autoimmune diseases. *Immunol. Lett.* 143 9–19. 10.1016/j.imlet.2012.01.013 22342996

[B39] Oen K. G. Cheang M. (1996). Epidemiology of chronic arthritis in childhood. *Semin. Arthritis Rheum* 26 575–591. 10.1016/S0049-0172(96)80009-6 8989803

[B40] Ombrello M. J. Arthur V. L. Remmers E. F. Hinks A. Tachmazidou I. Grom A. A. (2017). Genetic architecture distinguishes systemic juvenile idiopathic arthritis from other forms of juvenile idiopathic arthritis: clinical and therapeutic implications. *Ann. Rheumatic Dis.* 76 906–913. 10.1136/annrheumdis-2016-210324 27927641 PMC5530341

[B41] Onuora S. (2015). Rheumatoid arthritis: clues to the HLA-RA connection from T-cell crossreactivity to vinculin and microorganisms. *Nat. Rev. Rheumatol.* 11:384. 10.1038/nrrheum.2015.73 26009294

[B42] Orchard S. Kerrien S. Abbani S. Aranda B. Bhate J. Bidwell S. (2012). Protein interaction data curation: the International Molecular Exchange (IMEx) consortium. *Nat. Methods* 9 345–350. 10.1038/nmeth.1931 22453911 PMC3703241

[B43] Parkkola A. Laine A. P. Karhunen M. Harkonen T. Ryhanen S. J. Ilonen J. (2017). HLA and non-HLA genes and familial predisposition to autoimmune diseases in families with a child affected by type 1 diabetes. *PLoS One* 12:e0188402. 10.1371/journal.pone.0188402 29182645 PMC5705143

[B44] Petty R. E. Southwood T. R. Manners P. Baum J. Glass D. N. Goldenberg J. (2004). International league of associations for rheumatology classification of juvenile idiopathic arthritis: second revision, Edmonton, 2001. *J. Rheumatol.* 31 390–392. 14760812

[B45] Pons P. Latapy M. (2005). “Computing communities in large networks using random walks,” in *Computer and Information Sciences ISCIS 2005*, eds Yolum P. Güngör T. Gürgen F. Özturan C. (Berlin: Springer), 284–293. 10.1007/11569596_31

[B46] R Core Team (2018). *R: A Language and Environment for Statistical Computing.* Vienna: R Foundation for Statistical Computing.

[B47] Rao S. S. Huntley M. H. Durand N. C. Stamenova E. K. Bochkov I. D. Robinson J. T. (2014). A 3D map of the human genome at kilobase resolution reveals principles of chromatin looping. *Cell* 159 1665–1680. 10.1016/j.cell.2014.11.021 25497547 PMC5635824

[B48] Savolainen A. Saila H. Kotaniemi K. Kaipianen-Seppanen O. Leirisalo-Repo M. Aho K. (2000). Magnitude of the genetic component in juvenile idiopathic arthritis. *Ann. Rheum Dis.* 59:1001. 10.1136/ard.59.12.1001 11153478 PMC1753050

[B49] Schmitt A. D. Hu M. Jung I. Xu Z. Qiu Y. Tan C. L. (2016). A compendium of chromatin contact maps reveals spatially active regions in the human genome. *Cell Rep.* 17 2042–2059. 10.1016/j.celrep.2016.10.061 27851967 PMC5478386

[B50] Smemo S. Tena J. J. Kim K. H. Gamazon E. R. Sakabe N. J. Gomez-Marin C. (2014). Obesity-associated variants within FTO form long-range functional connections with IRX3. *Nature* 507 371–375. 10.1038/nature13138 24646999 PMC4113484

[B51] Smerdel A. Ploski R. Flato B. Musiej-Nowakowska E. Thorsby E. Forre O. (2002). Juvenile idiopathic arthritis (JIA) is primarily associated with HLA-DR8 but not DQ4 on the DR8-DQ4 haplotype. *Ann. Rheum Dis.* 61 354–357. 10.1136/ard.61.4.354 11874841 PMC1754045

[B52] Smolen J. S. Aletaha D. Barton A. Burmester G. R. Emery P. Firestein G. S. (2018). Rheumatoid arthritis. *Nat. Rev. Dis. Primers* 4:18001. 10.1038/nrdp.2018.1 29417936

[B53] Sollid L. M. (2017). The roles of MHC class II genes and post-translational modification in celiac disease. *Immunogenetics* 69 605–616. 10.1007/s00251-017-0985-7 28695286

[B54] Svantesson H. Bjorkhem G. Elborgh R. (1983). Cardiac involvement in juvenile rheumatoid arthritis. A follow-up study. *Acta Paediatr. Scand.* 72 345–350. 10.1111/j.1651-2227.1983.tb09726.x 6880720

[B55] Swaminathan B. Thorleifsson G. Joud M. Ali M. Johnsson E. Ajore R. (2015). Variants in ELL2 influencing immunoglobulin levels associate with multiple myeloma. *Nat. Commun.* 6:7213. 10.1038/ncomms8213 26007630 PMC4455110

[B56] Thompson S. D. Marion M. C. Sudman M. Ryan M. Tsoras M. Howard T. D. (2012). Genome-wide association analysis of juvenile idiopathic arthritis identifies a new susceptibility locus at chromosomal region 3q13. *Arthritis Rheum* 64 2781–2791. 10.1002/art.34429 22354554 PMC3366043

[B57] Van Ingen G. Li J. Goedegebure A. Pandey R. Li Y. R. March M. E. (2016). Genome-wide association study for acute otitis media in children identifies FNDC1 as disease contributing gene. *Nat. Commun.* 7:12792. 10.1038/ncomms12792 27677580 PMC5052699

[B58] Voskuyl A. E. (2006). The heart and cardiovascular manifestations in rheumatoid arthritis. *Rheumatology* 45(Suppl. 4), iv4–iv7. 10.1093/rheumatology/kel313 16980723

[B59] Wang Y. Song F. Zhang B. Zhang L. Xu J. Kuang D. (2018). The 3D genome browser: a web-based browser for visualizing 3D genome organization and long-range chromatin interactions. *Genome Biol.* 19:151. 10.1186/s13059-018-1519-9 30286773 PMC6172833

[B60] Ward L. D. Kellis M. (2012). HaploReg: a resource for exploring chromatin states, conservation, and regulatory motif alterations within sets of genetically linked variants. *Nucleic Acids Res.* 40 D930–D934. 10.1093/nar/gkr917 22064851 PMC3245002

[B61] Watanabe K. Taskesen E. Van Bochoven A. Posthuma D. (2017). Functional mapping and annotation of genetic associations with FUMA. *Nat. Commun.* 8:1826. 10.1038/s41467-017-01261-5 29184056 PMC5705698

[B62] Xia J. Benner M. J. Hancock R. E. (2014). NetworkAnalyst–integrative approaches for protein-protein interaction network analysis and visual exploration. *Nucleic Acids Res.* 42 W167–W174. 10.1093/nar/gku443 24861621 PMC4086107

[B63] Zhang X. Kluger Y. Nakayama Y. Poddar R. Whitney C. Detora A. (2004). Gene expression in mature neutrophils: early responses to inflammatory stimuli. *J. Leukoc Biol.* 75 358–372. 10.1189/jlb.0903412 14634056

