# Supplementary material for: Identification of Target Genes at Juvenile Idiopathic Arthritis GWAS Loci in Human Neutrophils
Source: Front Genet. 2019 Mar 27;10:181. doi: 10.3389/fgene.2019.00181 (PMC6445851; doi:10.3389/fgene.2019.00181)
Supplement: Supplementary file 1 [file Data_Sheet_1.pdf]

Identification of Target Genes at Juvenile Idiopathic Arthritis GWAS Loci in Human Neutrophils

Junyi Li, Xiucheng Yuan, Michael E. March, Xueming Yao, Yan Sun, Xiao Chang, Hakon Hakonarson, Qianghua Xia, Xinyi Meng, Jin Li

Supplementary Material

Supplementary Figure 1. JIA SNPs and target genes located in the same topologically associating domains (TAD).

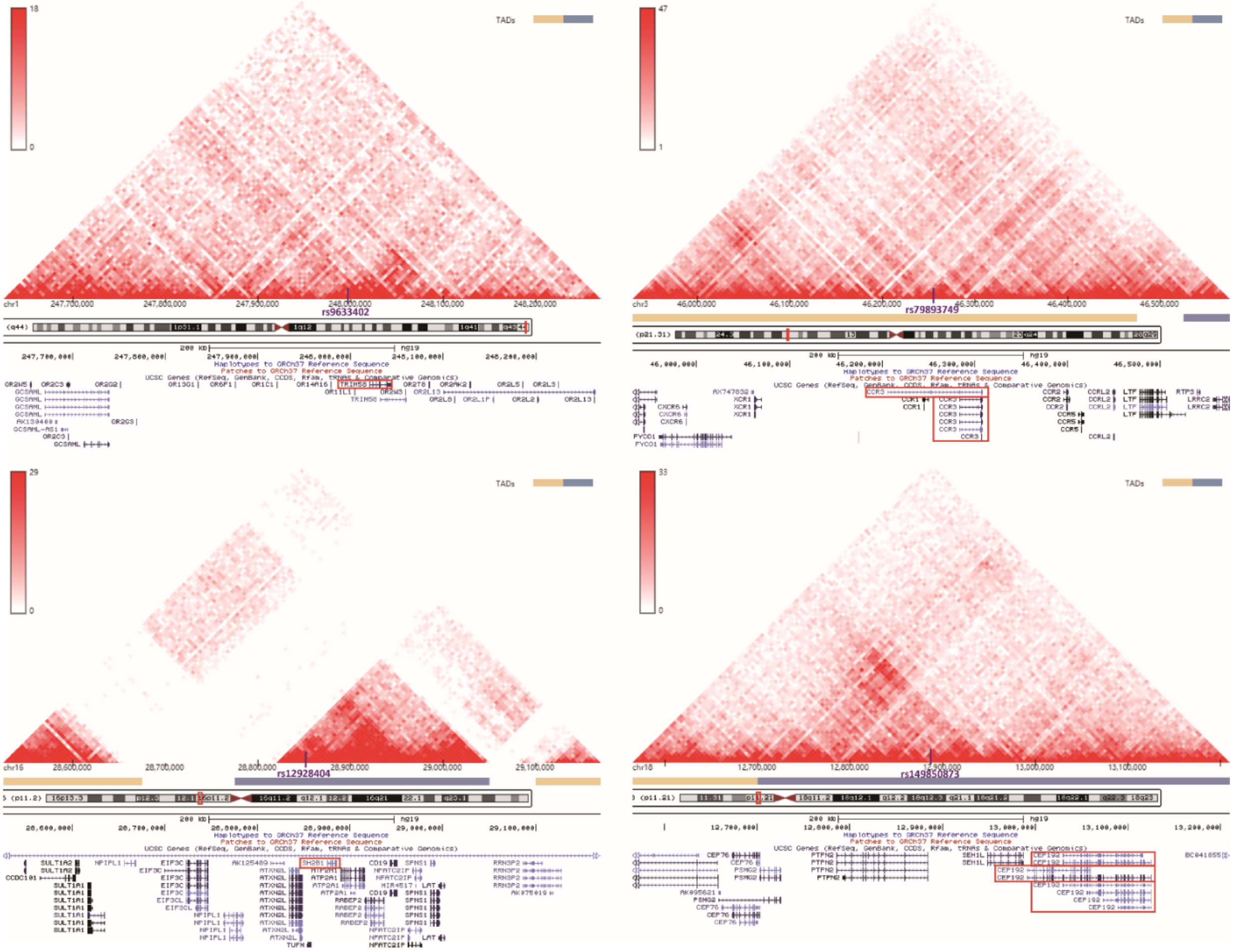

Supplementary Figure 2. Circos plots of chromatin interactions between JIA SNPs and target genes.

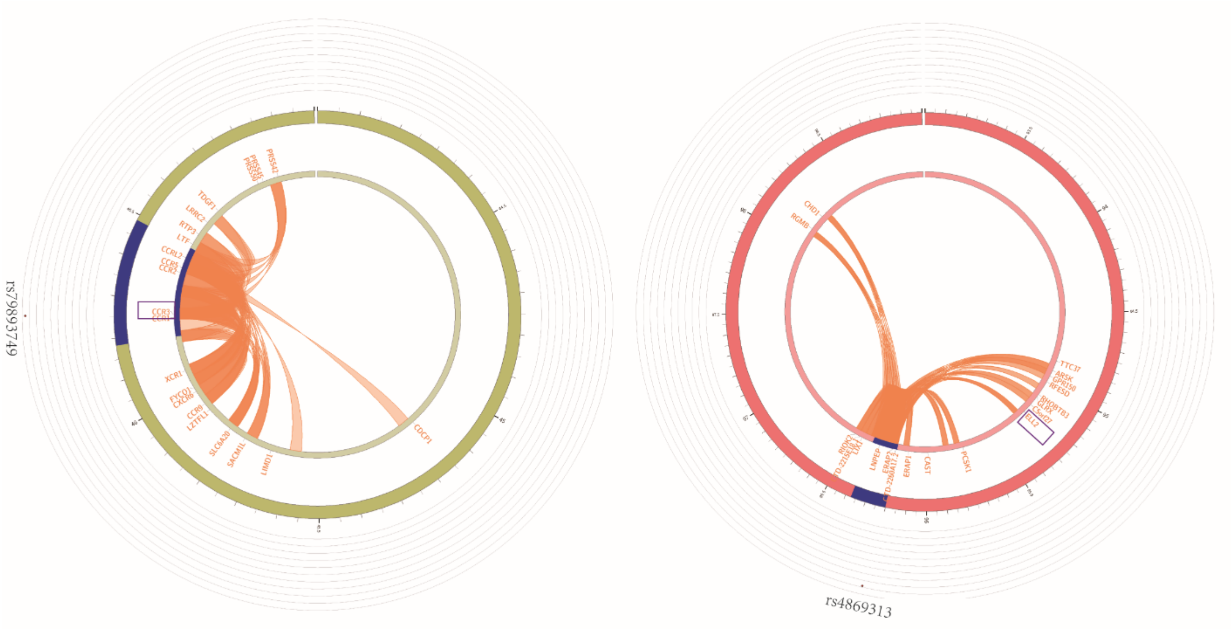

Supplementary Figure 3. PPI network of first-order interactions constructed for JIA loci target genes in PBMC.

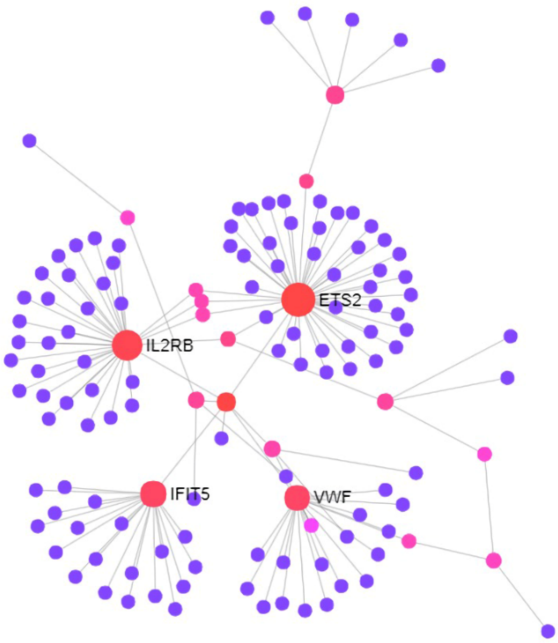

Supplementary Table 1. Summary of JIA GWAS loci targeted genes in PBMC

SNP	Chr	Pos (hg19)	Gene	GWAS_Pval	GTEx_Pval	DEG_PFP	DEG_FC
rs9633402	1	247946160	*TRIM58*	3.E-06	5.4E-04	6.70E-04	1.32
rs9633402	1	247946160	*ZNF124*	3.E-06	1.5E-02	1.77E-03	-1.32
rs2075184	2	103080592	*IL18RAP*	9.E-08	5.0E-34	8.30E-04	-1.42
rs2075184	2	103080592	*IL18R1*	9.E-08	1.0E-04	7.76E-03	-1.33
rs4676410	2	241563739	*FARP2*	2.E-07	1.8E-02	9.14E-03	-1.24
rs4625	3	49572140	*IP6K1*	*8.E-11	8.5E-03	2.68E-03	1.26
rs11741255	5	131811182	*SLC22A4*	*2.E-09	2.3E-02	5.56E-03	1.24
rs2395148	6	32321554	*HLA-DQB1*	*2.E-10	2.3E-03	3.33E-02	-1.28
rs41291794	6	32425762	*HLA-DPB1*	*4.E-15	2.1E-03	8.19E-04	-1.37
rs481331	10	43003048	*ZNF33B*	1.E-06	1.7E-12	1.36E-03	-1.33
rs7069750	10	90762376	*IFIT5*	*3.E-08	1.6E-04	2.80E-03	1.2
rs10849448	12	6493351	*VWF*	*5.E-09	4.8E-02	1.64E-05	1.35
rs12598357	16	28340945	*APOBR*	*4.E-09	3.7E-03	1.08E-02	1.22
rs2836882	21	40466570	*ETS2*	5.E-08	2.3E-08	6.85E-03	-1.27
rs10084630	22	37679487	*IL2RB*	9.E-06	5.8E-03	7.75E-03	-1.31

*SNP = single nucleotide polymorphism; Chr = chromosome; Pos (hg19) = position on human genome build hg19; Gene = likely targeted gene at each GWAS locus; GWAS_Pval =P-value of each SNP in GWAS analyses extracted from GWAS catalog; GTEx_Pval=P-value of correlation between gene expression level and GWAS SNP genotype in GTEx database; DEG_PFP = the probability of being false positive for the differentially expressed gene in the microarray analysis; DEG_FC = fold change of the differentially expressed gene in a microarray analysis; *GWAS_Pval < 5.E-08.*

Supplementary Table 2. KEGG pathways enriched in the PPI network formed by JIA loci target gene and their interactors in PBMC (FDR<0.05).

Pathway	Total	Expected	Hits	P-Value	FDR
Jak-STAT signaling pathway	99	1.92	20	8.74E-16	1.90E-13
Measles	102	1.98	16	5.35E-11	5.81E-09
HTLV-I infection	199	3.87	19	4.57E-09	3.31E-07
Pathways in cancer	310	6.03	20	1.25E-06	6.78E-05
Acute myeloid leukemia	57	1.11	8	1.14E-05	0.000493
Osteoclast differentiation	119	2.31	11	1.53E-05	0.000555
Prostate cancer	87	1.69	9	3.94E-05	0.00122
Renal cell carcinoma	60	1.17	7	0.000139	0.0036
Herpes simplex infection	103	2	9	0.000149	0.0036
ErbB signaling pathway	87	1.69	8	0.000249	0.00541
Pancreatic cancer	69	1.34	7	0.000337	0.00615
Epstein-Barr virus infection	91	1.77	8	0.00034	0.00615
Chronic myeloid leukemia	73	1.42	7	0.000478	0.00798
Influenza A	107	2.08	8	0.00101	0.0157
Dorso-ventral axis formation	12	0.233	3	0.00138	0.0199
Focal adhesion	200	3.89	11	0.00155	0.021
Wnt signaling pathway	144	2.8	9	0.00176	0.0222
Tuberculosis	174	3.38	10	0.00184	0.0222
Bladder cancer	29	0.564	4	0.00219	0.025
Colorectal cancer	49	0.953	5	0.00242	0.0262
Leishmaniasis	51	0.992	5	0.00289	0.0294
Hepatitis C	100	1.94	7	0.00307	0.0294
B cell receptor signaling pathway	75	1.46	6	0.00314	0.0294
Cytokine-cytokine receptor interaction	253	4.92	12	0.00331	0.0294
Chemokine signaling pathway	189	3.68	10	0.00339	0.0294
Transcriptional misregulation in cancer	19	0.369	3	0.0055	0.0459

Total = the total number of genes in each pathway; Expected = the expected number of genes in each pathway given the number of JIA target genes; Hits = the actual number of JIA target genes fall into each pathway; P-value = P-value of each pathway in enrichment test; FDR = false discovery rate of each pathway.
